# Hsp110 nucleotide exchange factors may amplify Hsp70-disaggregation by enhanced entropic pulling

**DOI:** 10.1016/j.jbc.2025.110450

**Published:** 2025-07-04

**Authors:** Mathieu E. Rebeaud, Bruno Fauvet, Paolo De Los Rios, Pierre Goloubinoff

**Affiliations:** 1 Institute of Physics, School of Basic Sciences, École Polytechnique Fédérale de Lausanne – EPFL, Lausanne, Switzerland; 2 Faculty of Biology and Medicine, Department of Plant Molecular Biology, University of Lausanne, Lausanne, Switzerland; 3 Institute of Bioengineering, School of Life Sciences, École Polytechnique Fédérale de Lausanne – EPFL, Lausanne, Switzerland

**Keywords:** Hsp110, Hsp70, entropic pulling, NEF, chaperones

## Abstract

Hsp70s use energy from ATP hydrolysis to unfold protein structures and solubilize stable aggregates and accumulate native species, even under adverse non-native conditions. To carry out its catalytic polypeptide-unfolding activity, Hsp70 needs to reversibly interact with a J-domain (JDP) catalyst, a misfolded or alternatively-folded polypeptide substrate and a nucleotide exchange factor (NEF), which binds to the nucleotide-binding domain (NBD), accelerates ADP-release, and controls the dissociation of the unfolded polypeptide product of the unfolding reaction. In bacteria, GrpE is the ubiquitous NEF, and yet, during the process of eukaryotization, it was lost from the cytosol, to be replaced by novel NEF proteins, among which the Hsp110 family stands out. Curiously, Hsp110s belong to the Hsp70 superfamily, but the evolutionary steps that led from an ancestral Hsp70 unfoldase to a Hsp110 NEF catalyzing other Hsp70's activity remain unsolved. Combining experiments using wild-type Sse1 (yeast Hsp110) and rationally designed mutants, we show that Hsp110 is likely built upon some distinctive features already present in Hsp70 by repurposing them, rather than by fashioning novel molecular properties. Taking all results together, we suggest a novel mechanism of action of Hsp110, whereby it is a NEF that also enhances the unfolding/disaggregating entropic pulling forces generated by Hsp70, by transiently increasing the chaperone's effective volume.

Genome analysis of simple and more complex free-living bacteria and archaea suggests that about three billion years ago, early terrestrial bacteria evolved the first Hsp70 chaperone from a preexisting actin/sugar hexokinase gene (1). Apart from very simple Hsp70-less archaea and bacteria, today, Hsp70s (DnaK in prokaryotes) are present in all the ATP-containing cellular compartments of cells. Members of the Hsp70 family may accumulate into being 1% of the total protein mass of eukaryotic cells (2). It acts as a polypeptide unfolding nanomachine that uses energy from ATP-hydrolysis to convert stable alternatively-folded, or misfolded polypeptides, into transiently unfolded intermediates, which upon chaperone dissociation, may spontaneously reach different conformations, such as the native state, even under non-native conditions favoring misfolding and aggregation (3, 4, 5, 6). Hsp70 co-evolved with obligate sub-stoichiometric (5–10 fold less) J-domain proteins (JDPs, sometimes also generically but inappropriately called DnaJs or Hsp40s), which accelerates ATP-hydrolysis and catalyzes Hsp70's binding to already misfolded or alternatively-folded polypeptides that are in need to be transiently unfolded, and with NEFs (Nucleotide Exchange Factors) that catalyzes ADP release (and ATP rebinding) and the consequent opening of Hsp70's protein binding lid, leading to the dissociation and native refolding of the polypeptide (7, 8). In complex bacteria and archaea, the DnaK-DnaJ-GrpE (KJE) system soon became the central hub of cellular protein homeostasis and, about a billion years later, it evolved and diversified in the cytosol and was further exported into the *endoplasmic reticulum* (ER) of the first eukaryotes. Today, in addition of unfolding protein aggregates, Hsp70-JDP systems regulate various key cellular functions necessitating structural shifts and changes of oligomeric states, affecting the biological activities of various native protein complexes (9, 10).

A phylogenetic analysis reveals that early in the process of eukaryotisation, an ancestral archaeal DnaK, which turned into being the first cytosolic Hsp70, did so while renouncing its billion-years dedicated nucleotide exchange factor (NEF) GrpE, for various new NEFs, such as HspBP1 (Fes1 in yeast) and Bag1 (Snl1 in yeast) (11), for which no obvious orthologous prokaryotic ancestor can be identified (12). Like GrpE, both NEFs don't hydrolyze ATP. Upon transiently binding to Hsp70's nucleotide-binding domain (NBD), both increase ADP-release and ATP binding and promote the release of the Hsp70-bound polypeptides (9). Importantly, the first eukaryotes (13) evolved a predominant new NEF family, called Hsp110, from an Hsp70 progenitor (DnaK), which became the most abundant NEF in the yeast and human cytosols. Soon after the appearance of the first cytosolic Hsp110, an orthologous version of it further evolved by gene duplication, to be exported and serve in the *endoplasmic reticulum* (ER) as the sole Hsp110 (called Lhs1 in yeast, Hyou1 or Grp170 in mammals) to the newly ER-exported Hsp70s (called BIPs) (12). About two billion years later, all Hsp110s in currently living eukaryotes are still highly homologous, sequence-wise and structurally, to their Hsp70 ancestor.

Here, we aimed to identify the transformations that changed an original Hsp70 chaperone, which was an ATP-fueled and JDP-dependent polypeptide-unfolding enzyme that binds NEFs, into Hsp110, which is an ATP-fueled and JDP-independent nucleotide exchange catalyst of Hsp70, apparently devoid of polypeptide-unfolding abilities by itself and that does not bind NEFs. Our approach was to use wild-type (WT) yeast Sse1 and some designed mutants, and measure the ATPase, prevention of aggregation, unfolding, disaggregation and refolding activities of Ssa1, Sis1, and Sse1, respectively, the most abundant Hsp70, JDP, and NEF cochaperones in the cytosol of yeast cells. We find that the Hsp110s have apparently renounced their ancestral ability to interact with, and have their ATPase activated by JDPs (14, 15). Without Hsp70s, Hsp110s have also apparently forsaken most of their ability to bind misfolded polypeptides, to interact with NEFs, and remodel various polypeptide substrates by ATP-fueled unfolding. Yet, the Hsp110s have apparently gained the ability to form transient heterodimers (16, 17) with Hsp70s through their conserved nucleotide binding domain (NBD) and, at variance with GrpE, Hsp110s oddly depend on ATP hydrolysis to accelerate ADP release from Hsp70 (18).

## Results

### Phylogenetic analysis of Hsp110s

Ssa1 and Sse1 are the most abundant Hsp70 and Hsp110 molecules in the cytosol of exponentially growing yeast, at 15 μM and 3 μM concentrations, respectively (19, 20, 21, 22). Ssa1 has three orthologues, Ssa2, Ssa3, and Ssa4, whose combined concentrations account for less than 4 μM, thus much less than Ssa1. The concentration of the Sse1 orthologue, Sse2, is about 0.3 μM, about ten times less than Sse1 (19, 20, 21, 22).

**Figure 1:**
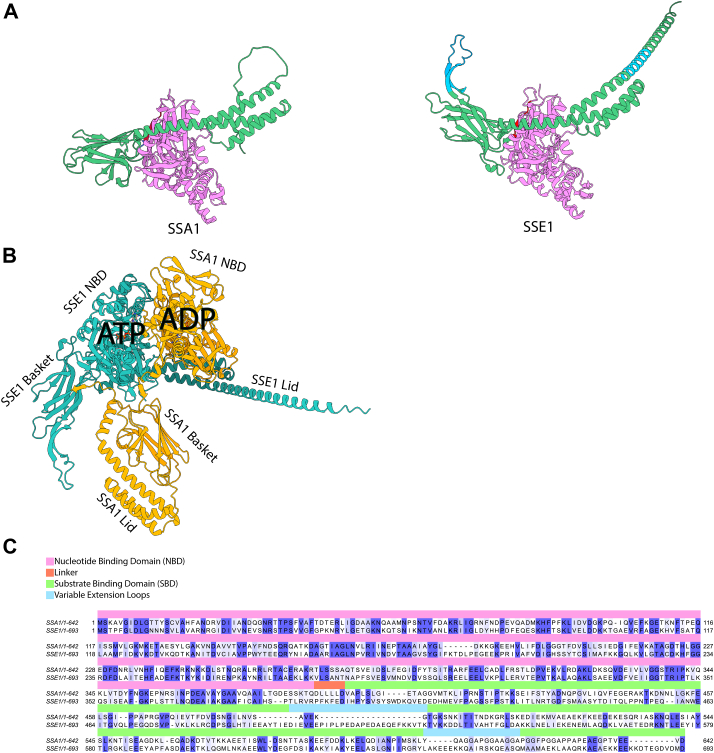
**Structures and sequences of yeast Ssa1 and Sse1.***A*, alphaFold (UNIPROT: AF-P32589-F1 and AF-P10591-F1) of ATP-bound Ssa1 (*left*) and ATP-bound Sse1 (*right*). Ssa1's Substrate Binding Domain (SBD, *Green*) is composed of a β-folded “basket” subdomain and an α-helical lid that can close onto a misfolded substrate. Non-homologous extended segments in Sse1's basket and lid are in *pale blue*. *B*, alphaFold *top* predicted model for an Ssa1-Sse1 heterodimer (*center*, Ssa1 in *orange* and Sse1 in *teal*) places ATP in Sse1 and ADP in a “relaxed” conformation of Ssa1, with a closed SBD that can potentially pull misfolded polypeptides out of a stable aggregate. In the AlphaFold top predicted model Ssa1 and Sse1 are found to be associated though their Nucleotide Binding Domain (NBD). *C*, amino acid sequence alignment of yeast SSA1 and SSE1 showing the conserved NBDs (*violet*), distinct likers (*red*) and highly variable extra-loops (*blue*) in the less conserved SBD (*green*). Amino acid conservation scores from 50 to 100% are highlighted in a *blue* gradient.

Structurally, Ssa1 and Sse1 are very similar (Fig. 1*A*), as expected for members of the same superfamily. Moreover, ADP-bound Hsp70 and ATP-bound Hsp110 can form heterodimeric complexes (23, 24), and AlphaFold three predictions (25, 26) confirmed preference for (ADP-Ssa1)-(ATP-Sse1) heterodimers (Fig. 1*B*). As a consequence, Sse1 is expected to preferentially bind ADP-bound, substrate-engaged Hsp70. The observed excess of Hsp70 over Hsp110 in the yeast cytosol implies in turn that while most Hsp110 molecules may be occupied by ADP-bound Hsp70 at any given time, the majority of free Hsp70 should preferentially be in the ATP-bound state, not to outcompete the substrate-bound form. Moreover, while a single Hsp110 binding/release cycle might be sufficient to complete the ATPase cycle of Hsp70, the driving of an optimal refolding activity of an excess of Hsp70 likely depends on multiple cycles of Hsp110 binding to and dissociating from Hsp70, suggesting highly dynamic Hsp70/Hsp110 heterodimers.

Sequence-wise, Ssa1 and Sse1 are highly homologous. Yet, Sse1 presents a distinctive linker between its NBD and SBD domains (PFKFED), which is a hallmark for yeast Hsp110s (27). This linker is clearly distinguishable from the highly conserved linker sequences of prokaryotic and eukaryotic Hsp70s, composed of a core of four small hydrophobic, non-aromatic residues, flanked by negatively charged ones (DLLLLD for Ssa1) (16, 28) (Fig. 1*C*). A phylogenetic tree of amino acid sequence alignments of Hsp110s from distant eukaryotes reproduced the known structure of the tree of life (Fig. S1*A*). The phylogenetic trees for the two separate domains confirmed that Hsp110's NBDs are much more conserved than their SBDs (Fig. S1, *B* and *C*). There are two highly variable regions in Hsp110's SBD, one in the so-called β-strand “basket”, and another in the α-helical “lid”, with unstructured extended segments, according to AlphaFold (Fig. S1). To simplify our search for what in general tells Hsp110s apart from Hsp70s, we chose Sse1 as a model Hsp110 because it has the shortest extensions in the SBD, while retaining all the other co-chaperoning features of the Hsp110s family.

It is possible that the evolutionary separation between Sse1 and Ssa1 is also due to the alteration of post-translational modifications (PTMs) sites (29). In GPMdb, there are 126 amino acids with PTMs on Ssa1 (69 in the NBD and 57 in the SBD) and 87 amino acids on Sse1 (50 in the NBD and 37 in the SBD). In this context, Sse1 appears to exhibit greater alterations at sites available for PTMs, considering the regulatory role of phosphorylation in Hsp70 client triaging, with Ssa1 and Sse1, possessing 76 and 48 phosphorylation sites, respectively.

### Sse1 by itself cannot prevent MlucV aggregation

Molecular chaperones are often thought to act as “holdases”, by way of binding stress-unfolded polypeptides, thereby preventing their further aggregation (9, 30). We monitored the individual and combined abilities of the main yeast cytosolic Hsp70 (Ssa1), JDPs (Sis1 and Ydj1) and NEF (Sse1), to prevent the aggregation of urea pre-unfolded MlucV (6). This reporter protein was previously developed to assess the native/non-native state of thermolabile mutated firefly luciferase (Luc) (31) by monitoring its enzymatic activity, and its degree of compactness by monitoring Förster Resonance Energy Transfer (FRET) between the N-terminal (M = mTFP1) and C-terminal (V = Venus) fluorescent proteins (6).

**Figure 2:**
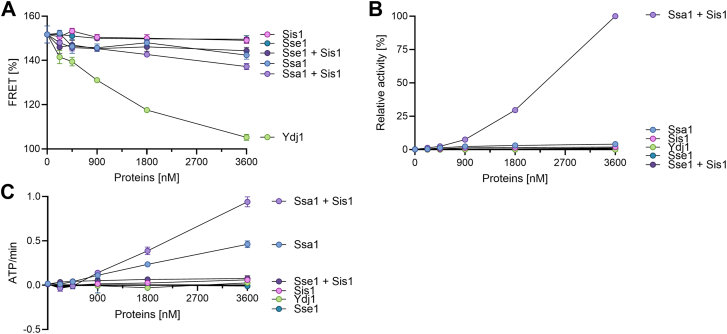
**The effect of increasing amounts of Ssa1 or Sse1, with MlucV aggregates, without or with Sis1 on ATP hydrolysis, the aggregation and the conversion of aggregates into native MlucV.** Urea-pre-unfolded MlucV was diluted in the presence of ATP and increasing concentrations of either Ssa1, or Sse1, without or with 900 nM Sis1. *A*, the FRET values of urea-pre-unfolded MlucV diluted in the presence ATP and increasing concentration of Ydj1, or Ssa1 or Sse1, without or with 900 nM Sis1. *B*, regain of native luciferase activity from urea-pre-unfolded MlucV, following 80 min in the presence of the different chaperone and co-chaperone mixes as in A. Maximum relative luciferase reactivation was set at 100% (Effective refolding yield of Ssa1+Sis1 being 5% of the maximal luciferase activity). *C*, ATPase activity of the different chaperones mixes as in A. In all panels, error bars represent mean ± SD (minimum of n = 3).

First, the luciferase core of MlucV was denatured in 4.8 M urea. Then, urea was extensively diluted by adding buffer and ATP, in the absence or presence of increasing amounts of Ssa1, without or with Sis1 and/or Ydj1 and/or Sse1 (Fig. 2*A*). After dilution, thus in conditions favoring the stability of the native state, in the absence of chaperones the luciferase core of MlucV was found to be and stay for a very long time enzymatically inactive, while the FRET values from the flanking urea-resistant native GFP-type fluorophores were typical of stable soluble MlucV aggregates, namely with about 150% of the FRET level set at 100% for the native MlucV (see (6)). When dilution and aggregation occurred in the presence of increasing amounts of Ydj1, a class A JDP similar to *Escherichia coli* DnaJ (32), the FRET signal was gradually lowered from 150% without Ydj1, to ∼105% at the largest Ydj1 concentrations. This is owing to the well-established ability of Ydj1 to bind polypeptides that misfold and thereby prevent their further aggregation (33, 34). In contrast, up to a 24 folds molar excess of Sis1, a class B JDP similar to *E. coli* CbpA (32), left the high FRET signal essentially unaltered, consistent with Sis1's recognized inability to bind unfolded/misfolding monomers and effectively prevent their aggregation (34). Like Sis1, Sse1 proved to be a very poor “holdase” by itself. Less expectedly, increasing amount of Ssa1, the canonical and most abundant cytosolic yeast Hsp70, only very mildly reduced the FRET signal of MlucV, down to ∼145% at the largest measured concentrations. In none of these cases the individual action of the chaperones and cochaperones subsequently resulted in a detectable recovery of the enzymatic luciferase activity, despite the presence of ATP in the assay (Fig. 2*B*), suggesting that the mild FRET reduction was associated with a very minor prevention of aggregation that did not subsequently lead to any refolded native species.

In the presence of constant (900 nM) Sis1 (that by itself, virtually does not prevent aggregation) and ATP, increasing amounts of Ssa1 or Sse1 had very different effects on urea-pre-unfolded, misfolding MlucV: Whereas both chaperone combinations reduced the FRET signal slightly more than the individual proteins (Fig. 2*A*), there was no recovery of luciferase activity by Sse1+Sis1 at any concentration while, in contrast, increasing concentrations of Ssa1 with Sis1 led to a mild but significant reduction of the FRET signal and a corresponding increment of the recovered luciferase activity (Fig. 2*C*), indicating that Sse1 lost Ssa1's ability to bind and be activated by JDPs. We also measured the ATPase activity of increasing concentrations of Sse1 or Ssa1, with aggregated MlucV, without and with Sis1 (Fig. 2*B*). Not surprisingly, the number of hydrolyzed ATP molecules per minute was undistinguishable from the background for Sis1 and Ydj1 alone, since they are not ATPases. Sse1, alone or in combination with Sis1, did not hydrolyze detectable amounts of ATP, despite having retained an intrinsic ATPase activity (14). By converse, increasing concentrations of Ssa1 showed a detectable ATPase activity, which doubled in the presence of Sis1.

### Hsp70s-JDPs have a significant disaggregase/refolding activity also without NEFs

In the presence of Sis1, Ssa1 weakly but effectively binds pre-aggregated polypeptides and uses energy from ATP-hydrolysis to dissolve them by unfolding by means of entropic pulling forces (6, 35, 36). According to this model, a NEF is expected to merely accelerate ADP dissociation and ATP rebinding, cause a consequent opening of Hsp70's SBD and allow the dissociation of the locally unfolded polypeptide intermediate, which can freely refold in solution, ultimately to the native state (3, 6, 37, 38). Given the purported NEF function of Sse1, we next addressed its functional effects on the protein disaggregation and refolding activity of the corresponding Hsp70-JDP chaperone system (Ssa1-Sis1) and compared it with the bacterial Hsp70-JDP-NEF system (DnaK-DnaJ-GrpE, KJE).

**Figure 3:**
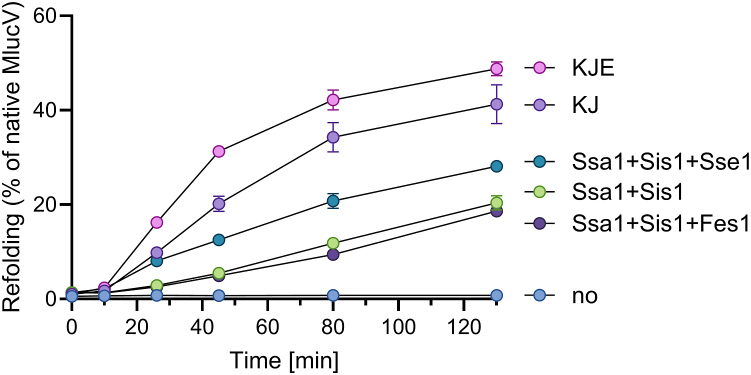
**Bacterial and yeast HSP70-JDP can disaggregate and refold stable aggregates in the absence of NEFs.** Time-dependent regain of luciferase activity from pre-aggregated MlucV, by yeast or *E. coli* chaperones: 150 nM of stable, inactive MlucV aggregates were incubated up to 120 min at 25 °C in the presence of 5 mM ATP, 4000 nM Hsp70 (DnaK or Ssa1), 1000 nM JDP (Sis1 or DnaJ), with or without NEF (500 nM Sse1, 1000 nM Fes1 or 1000 nM GrpE), as indicated. In all panels, error bars represent mean ± SD (n = 3).

While urea-pre-unfolded and preaggregated MlucV species did not spontaneously revert to native MlucV over time (Fig. 3), the bacterial KJ system without GrpE, together with ATP, already effectively converted a large fraction of the stable MlucV aggregates into native MlucV. Having GrpE present (KJE) further enhanced the MlucV reactivation rates and yields, albeit only mildly.

Similarly, but at about half the rate of the bacterial chaperones, Ssa1 together with Sis1 but without Sse1 also effectively converted a significant fraction of the stable aggregates into native MlucV. Addition of an optimal concentration of Sse1 (500 nM, in the same Ssa1/Sse1ratio as the yeast cytosol) mildly but significantly increased the luciferase refolding rates and yields. Addition of Fes1, another NEF in the yeast cytosol, did not stimulate the intrinsic disaggregase/refolding activity by Ssa1+Sis1 alone, an effect that at the tested concentration was thus specific to Sse1. Contrary to the obligate need for a JDP co-chaperone, without which Hsp70 had no refolding activity, both prokaryotic GrpE and eukaryotic Sse1 thus appear, *in vitro*, to be less obligate cochaperones: their action merely accelerated to a minor extent the refolding rates and increased the refolding yields.

**Figure 4:**
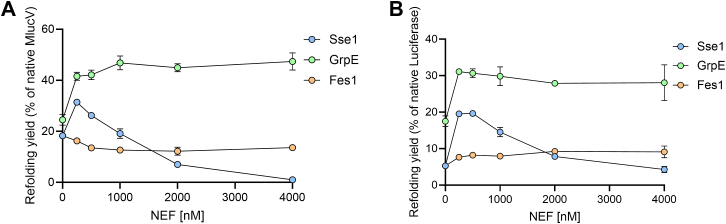
**Dose-responses of bacterial and eukaryotic NEFs with different substrates.***A*, MlucV was preaggregated (150 nM) in the presence of 4 mM ATP, 4000 nM Ssa1 and 1000 nM Sis1 and increasing amounts of Sse1. For comparison, the same reaction was carried with 4000 nM DnaK, 1000 nM DnaJ and increasing amounts of GrpE. *B*, similar high yields were observed as in A but using stable inactive luciferase aggregates (without fluorophores), which form much larger and more compact insoluble oligomers than stable MlucV aggregates (6, 66). In all panels, *error bars* represent mean ± SD (n = 3).

We next performed a dose-response of the NEFs and measured the luciferase reactivation of pre-aggregated MlucV and luciferase after 2 h of chaperone action in the presence of increasing NEF concentrations. We confirmed that both DnaK-DnaJ and Ssa1-Sis1 have stand-alone disaggregation and refolding activity of their own, which does not require the presence either of GrpE nor of Sse1, respectively. This notwithstanding, both NEFs enhanced the activity of their corresponding chaperone systems: Increasing amounts of GrpE nearly doubled the yields of the stand-alone disaggregation and refolding reaction by DnaK-DnaJ, with an EC_50_ at about 200 nM, corresponding to ∼40 times fewer GrpE dimers than DnaK monomers (Fig. 4*A*). This is suggestive of a very dynamical mode of action, where for an optimal activation of DnaK-DnaJ, a single GrpE dimer may iteratively bind, activate and dissociate from as many as 20 different DnaK molecules. Sse1 similarly showed a dynamical mode of action, since it nearly doubled the yields of the stand-alone disaggregation and refolding activity by Ssa1-Sis1, with an EC_50_ at 100 nM, when Sse1 was ∼40 times less than Ssa1 (Fig. 4, *A* and *B*). At concentrations higher than 250 nM, Sse1 increasingly inhibited the disaggregation and refolding reaction, with an IC_50_ at 1200 nM and a complete inhibition at the non-physiological, equimolar concentrations of Sse1 and Ssa1. Similarly, equimolar human Apg2 has been reported to strongly inhibit Hsc70 (39, 40, 41). Mild inhibition has been previously reported also for GrpE, albeit only when in excess over DnaK. At variance, 2 to 4 times less Sse1 than Ssa1 was shown to already inhibit the reaction (42).

**Figure 5:**
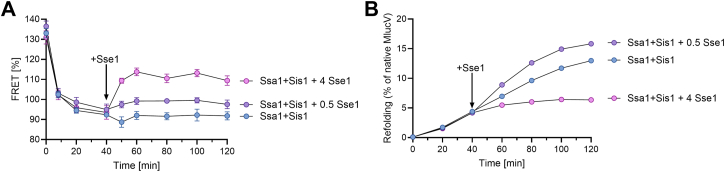
**Excess Sse1 prevents unfolding by Ssa1-Sis1, arrests native refolding and causes aggregation.** MlucV was pre-unfolded in urea and at T = 0′, was abruptly diluted 100-fold in refolding buffer containing 4 mM ATP. At T = 5′ min, 4000 nM Ssa1 and 500 nM Sis1 were added. At T = 40′, 500, 4000 nM or no Sse1 was added to the mix as indicated. FRET signals (*A*) and luciferase activity (*B*) were measured for 2 h at 25 °C. In all panels, error bars represent mean ± SD (n = 3).

To address the process by which the stand-alone disaggregation and refolding reaction by Ssa1-Sis1 becomes inhibited by an excess of Sse1, we next measured the time-dependent changes of the FRET value and of the recovered luciferase activities of preaggregated MlucV. When incubated at 34 °C with Ssa1 and Sis1 in the presence of ATP, a sharp decrease of the FRET signal of the preaggregated MlucV was observed (Fig. 5*A*), which confirmed that even without Sse1 the initial addition of Ssa1 and its JDP cochaperone can effectively drive the decompaction and unfolding of the stable preaggregated MlucV species and lead to the recovery of native luciferase activity (Fig. 5*B*). Subsequent addition (t = 40 min) of Sse1 at a concentration close to the optimal ratio with Ssa1 (500 nM, see Fig. 4), nearly doubled the rate of MlucV refolding, and to a lesser extent the yields. As expected from the accumulation of more native MlucV, the FRET signal also slightly increased because of the conversion of low-FRET chaperone-bound unfolded species into more compact native species with a higher FRET signal (Fig. 5*A*) (6). In contrast, the addition of a large, non-physiological excess of Sse1 (4000 nM, equimolar to Ssa1) abruptly increased the FRET signal (Fig. 5*A*), Correspondingly, the luciferase reactivation of MlucV was also halted (Fig. 5*B*). This is suggesting that when excessively bound by Sse1, Ssa1 is not only unable to convert bound MlucV to a fully unfolded conformation but that instead favored the aggregation of incompletely unfolded, prematurely released, misfolded MlucV species.

Similar results were obtained in the absence or presence of YS, a dysregulated hyper-active J-domain co-chaperone of Ssa1 (34). YS is a chimeric protein comprising the J-domain of Ydj1 and the G/F linker and CTDs of Sis1. The swap of the J-domain disrupts the auto-inhibitory interactions between the two parts, making the J-domain always available for Ssa1 binding. Like Sis1, with which it shares the CTDs, YS was unable by itself to prevent MlucV aggregation (Fig. S2*A*). With ATP, the addition of 4000 nM Ssa1 to YS caused a decrease of initial FRET value and a mild, yet significant luciferase refolding was measured (Fig. S2*B*). This indicated that most of the preaggregated substrates that was readily bound to Ssa1, was prevented from further aggregating and with ATP, was unfolded and subsequently released to natively refold. Expectedly, the time-dependent unfolding and native refolding was increased with the addition of an optimal, sub-stoichiometric amount of Sse1 (500 nM). Instead, and in line with the results in Fig. 5, excess equimolar Sse1 abolished the ability of the Ssa1 and YS to prevent MlucV aggregation and/or to unfold and disaggregate preformed aggregates and subsequently lead to their native refolding.

### Nucleotide exchange factors rescue Hsp70 activity at high ADP concentrations

Despite our *in vitro* activity assays indicating that GrpE is a less essential co-chaperone than DnaJ for DnaK, in bacteria GrpE is often found associated with DnaK and DnaJ in the same operon (43), suggestive of its very important primordial function. Similarly, Sse1 is constitutively expressed in the cytosol of yeast. Whereas in some extreme cases, like for the *in vitro* disassembly of compact α-synuclein fibrils, Hsp110s seem to be stringently essential (44), our results with less compact aggregates show that in general, NEFs rather play the role of optimizing the strong stand-alone disaggregation and refolding activities of Hsp70s-JDPs core-machinery. To further investigate the degree of essentiality of NEF cochaperones in bacteria and yeast, we next addressed their effect in the presence of limiting ATP and increasing ADP concentrations. Indeed, from their name, the putative function of NEFs is to facilitate the release of ADP from Hsp70 and the consequent rebinding of a new nucleotide molecule, which most likely would be ATP in the usual ATP excess conditions, but might be increasingly ADP as ATP concentration decreases, for example, in energy-depleted cells. It is important to stress that in quiescent micro-organisms, as well as under starvation, the ADP/ATP ratios can be elevated (45, 46).

There are two reasons to expect that increasing the ADP concentration relative to ATP should inhibit the Hsp70-JDP system. First, ADP locks Hsp70 in its closed state, preventing substrate release and subsequent native refolding and rebinding of a new misfolded substrate. Second, the energy available from ATP hydrolysis, which powers the chaperone machinery, is given by

$$\Delta G_{\text{ATP}}=k_{B}T\;\ln\left\{\left(\frac{\left[\text{ATP}\right]}{\left[\text{ADP}\right]\left[\text{Pi}\right]}\right)/{\left(\frac{\left[\text{ATP}\right]}{\left[\text{ADP}\right]\left[\text{Pi}\right]}\right)}_{eq}\right\}$$

where $\left(\frac{\left[\text{ATP}\right]}{\left[\text{ADP}\right]\left[\text{Pi}\right]}\right)$ is the concentration ratio of ATP to its hydrolysis products in the chaperone assay, and ${\left(\frac{\left[\text{ATP}\right]}{\left[\text{ADP}\right]\left[\text{Pi}\right]}\right)}_{eq}$ is the equilibrium ratio, if the nucleotide solution were allowed to reach thermodynamic equilibrium ([ADP]≫[ATP]). Increasing ADP amounts reduce the first ratio, thereby depriving Hsp70 of the energy required for its function.

**Figure 6:**
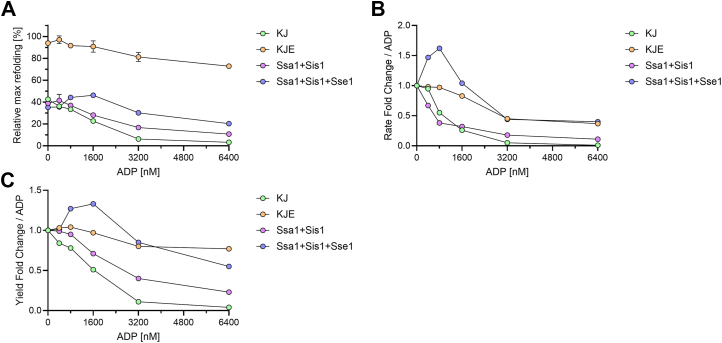
**NEFs reduce inhibition by excess ADP of prokaryotic and eukaryotic Hsp70s.***A*, 150 nM MlucV was pre-aggregated and incubated with 800 nM ATP, 6000 nM Ssa1 (or DnaK), 1000 nM Sis1 (or DnaJ) and increasing ADP, without or with 800 nM Sse1 (or GrpE). Recovered luciferase activity was measured after 3 h. The 100% relative maximal refolding fixed on the highest value corresponds to around 40% of native MlucV. *B*, without and with Sse1, equimolar ADP respectively inhibits and activates Ssa1+JDP-mediated disaggregation and refolding of pre-aggregated MlucV. Optimal Luciferase refolding rates from pre-aggregated MlucV as in A, incubated at 30 ^o^C for up to 3 h with 800 nM ATP, 6000 nM Ssa1, 1000 nM Sis1,1000 nM Ydj1 and increasing concentrations of ADP, without (*blue*), or with 800 nM Sse1. *C*, same as *B* but for the yields. In all panels, *error bars* represent mean ± SD (n = 3).

We thus measured the ability of either DnaK or Ssa1, in the presence of their respective JDPs and increasing amounts of ADP over a fixed near-limiting amount of ATP (0.8 mM), to natively refold stable preaggregated inactive MlucV species over time, without or with GrpE or Sse1, respectively (Fig. 6). Expectedly, in the absence of NEFs, both the prokaryotic and eukaryotic Hsp70-JDP systems were progressively inhibited by higher ADP concentrations over a constant limiting concentration of ATP (800 nM), with an IC_50_ for [ADP]/[ATP]≈2 (Fig. 6*A*). More surprisingly, the presence of sub-stoichiometric amounts of NEFs (3 and 7.5 times less in the case of GrpE and Sse1, respectively), significantly alleviated the inhibition by an excess of ADP over ATP. Remarkably, in the case of Ssa1-Sis1 with Sse1, equimolar ADP and ATP caused a slight, but reproducible, increase in the refolding yields (Fig. 6, *A* and *C*) and rates (Fig. 6*B*).

### The role of ATP hydrolysis for Sse1 function

The Hsp110 co-chaperone family exhibits significant divergence from the Hsp70s from which it derived, particularly in the so-called substrate-binding domain (SBD) and in the linker, which in the Hsp70s specifically interacts with the conserved HPD motive of J-domain protein co-chaperones. The SBDs and linkers of the Hsp110s also strongly repress ATP hydrolysis in their NBDs, more tightly than they do in Hsp70 (14, 47, 48, 49). At variance with its Hsp70 ancestor, Sse1 has apparently lost the ability to interact with and be activated by JDPs (as also predicted by AlphaFold protein complex predictions, Fig. S3), and compared to Ssa1, it has a greatly reduced ability to bind misfolded luciferase, both directly and indirectly, by way of JDP-mediated substrate-binding (Fig. 2*A*).

Despite these observations, the preservation in the course of two billion years of evolution of the ATPase activity of Sse1 suggests that ATP hydrolysis still carries a central role in the ability of Sse1 to boost the basal stand-alone disaggregation mechanism of the Hsp70-JDP system, even though it is not clear exactly under which condition it is necessary, as several studies showed contradictory results for the necessity of Hsp110 ATPase activity (50). We thus compared the action of increasing concentrations of WT Sse1 to that of the known Sse1-K69M mutant (51, 52, 53), which is considered to be ATPase-defective because its isolated K69M-NBD hydrolyzes ATP ∼15 times slower than isolated WT-NBD of Sse1 (14).

**Figure 7:**
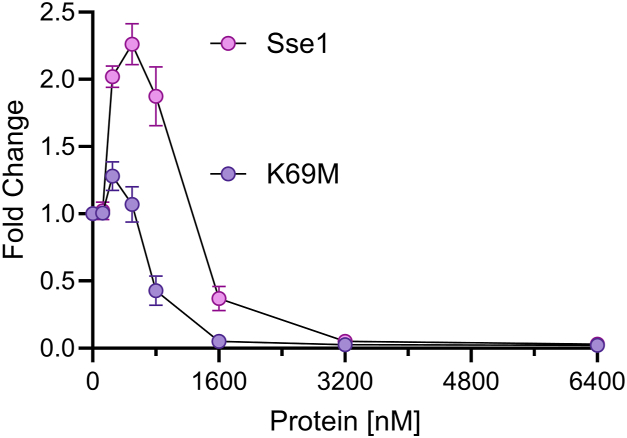
**Fold change of refolding between Sse1 and Sse1-K69M.** MlucV refolding yields of pre-aggregated Luciferase (200 nM) incubated for 2-h with 6000 nM Ssa1 and 1000 nM Sis1 in the presence of increasing concentrations of wild-type Sse1 or the K69M mutant. Luciferase activity yields are expressed as fold change from the basal, stand-alone activity of 6000 nM Ssa1 and 1000 nM Sis1. In all panels, *error bars* represent mean ± SD (n = 3).

Disaggregation assays showed that K69M activated significantly, even if only slightly, the Ssa1-driven disaggregation/refolding of pre-aggregated luciferase at an optimal concentration of 200 nM, which is 3 times lower than for WT Sse1 (600 nM) (Fig. 7). Correspondingly, inhibition by excess K69M Sse1 occurred at a concentration four times lower than for WT Sse1, suggesting that the K69M mutant associates more strongly with Ssa1, and dissociates from it more slowly than WT Sse1. These data hint at ATP hydrolysis being essential to drive an obligatory dissociation step of the Ssa1-Sse1 heterodimer, whose role is yet unclear for the effective Hsp70-JDP-mediated disaggregation cycle.

### Disinhibition of Sse1's ATPase does not correlate with the activation of disaggregation

The linker between the NBD and SBD domains of Hsp70 chaperones is highly conserved across the different kingdoms of life (DLLLLD in Ssa1, with relatively small variations, *e.g.,* DVLLLD in DnaK) (28). It specifically interacts with the highly conserved HPD motif of the J-domain in the crystal structure of the ATP-bound Hsp70-J-domain complex (37), while it should not interact with it in the ADP-bound Hsp70, when it is undocked from the NBD and completely extended and exposed to the solvent. The sequence of the linker is very different in Hsp110s (PFKFED in Sse1) (27) and most likely does not interact with J-domains (54), as also evidenced by the lack of Sse1's ATPase activation by JDPs (14) and by the lack of J-domain/NBD interaction in the AlphaFold three predicted structure of the Sse1/J-domain (of Sis1) complex (by comparison, the structure of the Ssa1/J-domain complex perfectly reproduces the DnaK/J-domain known complex) (7) (Sup Fig. 3). The linker thus serves as a major hallmark distinguishing Hsp70s from Hsp110s (28, 48). Furthermore, there is a complex interplay between the ATPase activity of Hsp70 and the domain interactions between NBD and SBD and with the linker: docking of the proximal part of the SBD's α-helix on the NBD inhibits ATP hydrolysis, whereas docking of the flexible linker on the NBD accelerates ATP hydrolysis (55). We thus designed two Sse1 mutants that, according to the findings for DnaK, could change the ATPase activity of Sse1.

**Figure 8:**
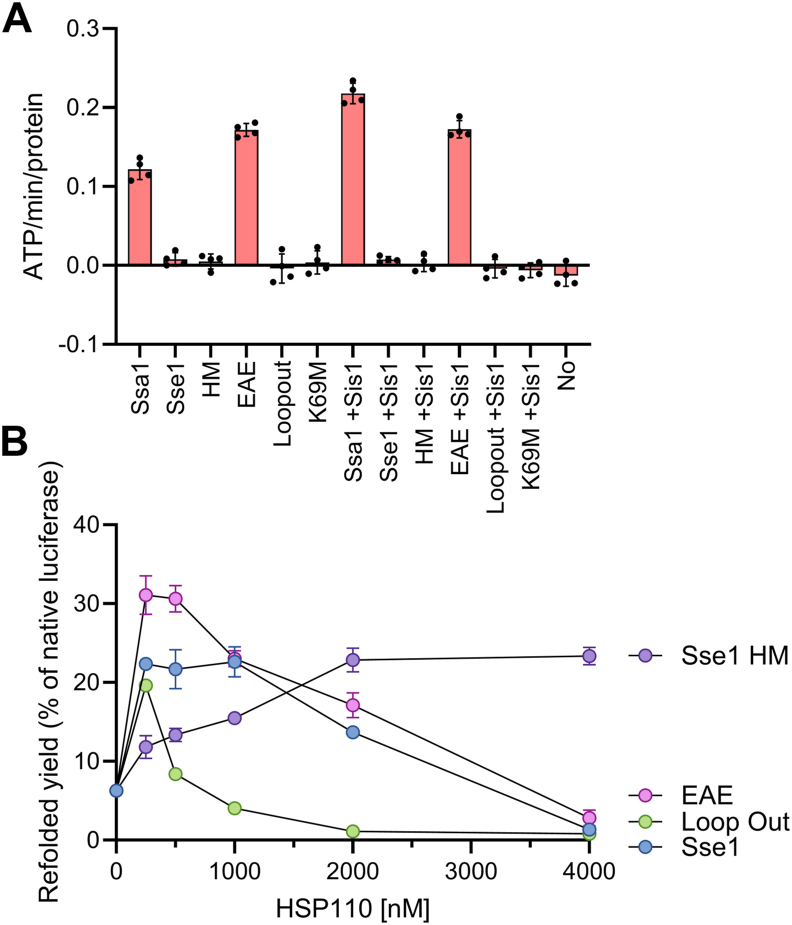
**ATPase and activation profiles of Sse1 mutants on Ssa1-Sis1 disaggregation.***A*, the rate of ATP hydrolysis measured for 60 min at RT, in the absence or presence of 6000 nM Ssa1or WT Sse1, K69M, EAE, HM or Loopout mutants, in the absence or presence of 1000 nM Sis1. *B*, luciferase refolding yields of 200 nM preaggregated Luciferase following incubation for 180 min at RT, in the presence of 6000 nM Ssa1, 1000 nM Sis1 and increasing concentrations of WT Sse1, K69M, EAE, HM or Loopout mutants. In all panels, *error bars* represent mean ± SD (minimum of n = 3).

In one mutant (Sse1-EAE), the PFKFED linker segment of Sse1 was changed into the DLLLLD linker from Ssa1 (Figs. S4 and S5). Compared to WT Sse1, the ATPase activity of EAE (without substrate) was strongly enhanced (Fig. 8*A*), to a level comparable to the activity of Ssa1. Yet, at variance with Ssa1, EAE's ATPase activity was equally high with and without Sis1. This indicates that in WT Sse1 the ATP hydrolysis is strongly repressed both by the PFKFED linker and by other parts of the SBD and becomes partially de-repressed when replaced by the Hsp70's linker segment. Yet, the replacement of Hsp110's linker by Hsp70's did not suffice to restore Sse1's ability to bind Sis1 and to further activate its ATPase, nor did it restore Sse1's ability to bind and process aggregates. We then tested the ability of increasing doses of EAE to facilitate protein disaggregation by Ssa1 in the presence of Sis1 and found that it recapitulated the same behavior of WT Sse1, with sub-stoichiometric amounts optimally activating, and stoichiometric amounts strongly inhibiting Ssa1-Sis1-mediated disaggregation and refolding (Fig. 8*B*). Thus, although more wasteful in ATP, EAE was as potent a co-disaggregase NEF as WT Sse1, and its mildly unleashed constitutive ATPase activity did not change the degree of inhibition by excess Sse1 binding to Ssa1.

To investigate the effect of the docking of other parts of Sse1's SBD, of the α-helical lid in particular, on ATP hydrolysis in the NBD, we designed a second Sse1 mutant, Sse1-HM, in which the proximal part of the helical lid of Sse1 was exchanged for the corresponding helical segment from Ssa1 (37, 49) (Fig. S5). Since this segment did not co-evolve with Sse1's NBD, we expected it to bind less tightly, and thereby possibly perturb, the strong repression by the lid of the ATPase activity. Yet, the results showed that ATP hydrolysis in the HM mutant remained as tightly auto-repressed as in WT Sse1 (Fig. 8*A*), suggesting that other regions of the SBD contribute to Sse1's ATPase autorepression. In contrast, we found that the HM mutation reduced the affinity of Sse1 for Ssa1 such that five times more HM mutant was necessary to half activate the refolding reaction (EC_50_ = 500 nM instead of 100 nM), and equimolar HM mutant did not inhibit the Ssa1-Sis1-mediated disaggregation and refolding reaction. This suggests that while the part of the helical lid of Sse1 that we swapped is likely not involved in the ATPase autorepression, it could be implicated in the regulation of its ATPase cycle by the interaction with Ssa1, and by the Ssa1 conformational changes that take place upon nucleotide exchange.

### The role of loop extensions in Sse1's SBD

A hallmark of all Hsp110s is the presence of loop extensions with unclear, possibly unfolded structures, in the SBD β-basket and the C-terminal end of the lid. Sse1 is no exception, although the length of the extra loops is very limited compared to plant and metazoan homologues. To explore the role of these extensions, we generated the Sse1 Loopout mutant, where one loop and one part of the helical lid have been removed (Figs. S4 and S5). We tested the effect of the Loopout mutations on the intrinsic ATPase activity and the effect of increasing concentrations of Sse1 Loopout on its ability to boost disaggregation by Ssa1-Sis1. Because the Loopout mutant has a wild-type NBD, a canonical Sse1 linker, and the extensions are not supposed to have interactions with the NBD, we expectedly found that the ATPase activity of Loopout was similarly very low, auto-repressed, as in the WT Sse1 (Fig. 8*A*). Surprisingly though, the Loopout mutant was found to activate the disaggregation action of Ssa1-Sis1 similarly to the K69M hydrolysis-deficient mutant *i.e.* it enhanced disaggregation almost as much as Sse1, albeit at smaller concentrations (up to 125 nM), and excess was inhibitory at lower concentrations than for WT Sse1, with an IC_50_ at about 500 nM (Fig. 8*B*, S6). This suggests that to boost the Ssa1-JDP disaggregation cycle, Sse1's extra-loops might favor, somehow, the obligatory dissociation from Ssa1 of Sse1, which requires ATP hydrolysis by Sse1 and is otherwise tightly auto-repressed in the other phases of the cycle.

## Discussion

The presence of Hsp110 in the genome of all eukaryotes and its functional role raises two intersecting questions: (i) What is their molecular mechanism of action? (ii) What are the evolutionary steps that have transformed an ancestral Hsp70 into a Nucleotide Exchange Factor? We can break these questions into different sub-issues.

### Hsp70-Hsp110 interactions

Activation and inhibition of Hsp70 in a dose-dependent manner clearly hint at an effect due to a direct interaction between Hsp70 and Hsp110, which we propose here, in keeping with previous works (56, 57), to have evolved from a pre-existing Hsp70-Hsp70 interaction template. Indeed, the contacts between the NBDs of the two proteins, as observed both in their crystal structures (23, 24) and in AlphaFold three predictions (54), mirror the overall NBD-NBD arrangement found in the Hsp70 homodimer crystal structure (58, 59, 60). The question is thus whether the Hsp70-Hsp110 heterodimer also inherited the function(s) of the Hsp70 homodimer or if, in the course of evolution, it has switched to a different role. Unfortunately, the function of the Hsp70 homodimer is not yet unambiguously known, despite being likely important due to its evolutionary conservation (61), and despite the observation that weakening the inter-protomer interactions causes defects in the function of DnaK (56, 57, 60, 62). We propose here that, prior to the emergence of Hsp110 as a dedicated NEF, identical Hsp70 molecules may have already partially fulfilled this function for one another: the binding of an ATP-bound Hsp70 to an ADP-bound Hsp70 might induce ADP release and subsequent ATP binding because of the high preference of the Hsp70 homodimer for ATP bound to both protomers. Of course, we cannot exclude the possibility that the transformation of an ancient Hsp70 into an Hsp110 NEF constitutes a genuine “gain of function.” Future targeted experiments are needed to test which of these two hypotheses is correct.

### Hsp110 ATPase activity

The enhancement of Hsp70's disaggregation and refolding activity occurs at low concentrations of Hsp110. This suggests that Hsp110 preferentially targets the limited pool of substrate-bound, ADP-bound Hsp70, rather than the excess of ATP-bound Hsp70 present in solution. Such specificity would imply that Hsp110 must have a very high affinity for the ADP-bound form of Hsp70—an affinity that must be rapidly downregulated after ADP-to-ATP nucleotide exchange in Hsp70. The data for the K69M mutant, which inhibits Hsp70 at lower concentrations compared to WT Sse1, suggest that the ATPase activity of Hsp110 is used to change its state into one with lower affinity for ATP-bound Hsp70. In this scenario, at high concentrations, Sse1 would also bind free ATP-bound Hsp70s despite its lower affinity with respect to ADP-bound, substrate-bound Ssa1. The enhanced bulkiness of these complexes would hinder their binding to aggregates, thus reducing their disaggregation activity. Yet, thanks to its ATPase activity, the Hsp110 affinity for ATP-bound Hsp70 would be reduced, limiting this counterproductive effect. Because of its much slower ATPase, K69M would instead retain a significant affinity for ATP-bound Hsp70 in solution, thus resulting in an inhibition of disaggregation, even at lower concentrations. From an evolutionary perspective, this proposal points to a functional repurposing, rather than a loss, of the ancestral Hsp70 ATPase, which has been conserved for over two billion years of Hsp110 evolution.

The inhibition of substrate refolding at high Hsp110 concentrations could alternatively be explained by the ability of the NEF to induce the dissociation of ATP (51), thus switching Hsp70 into the nucleotide-free state that is less prone to bind substrates. Yet, the K69M mutant induces ATP release roughly at the same rate as the wild-type, so explaining the difference in their inhibitory effect at high concentrations might be difficult to explain according to this view. More work is needed in the future to settle this issue.

### Substrate (Hsp70) sensing

Just as ATP-bound Hsp70s are informed by an allosteric signal when to hydrolyze ATP through their in-built SBD sensor for substrate binding and through the interaction of their highly conserved linker with the HPD motif of J-domain cochaperones, one can also expect Hsp110 to be informed by an allosteric signal when to hydrolyze ATP, when the bound Hsp70 undergoes nucleotide exchange. The EAE and Loopout mutants helped shed light on this issue.

EAE had an intrinsic ATPase rate of 0.17 ATP min^-1^, much higher than the wild-type Sse1, but their disaggregation/refolding activity was similar. It is tempting to propose that the wild-type linker has been specifically selected by evolution to avoid interaction with the HPD conserved triad of J-domains and thus eliminate a spurious interaction without affecting the rest of the function. The Loopout mutant, instead, affected the action of Ssa1 similar to the ATPase-deficient K69M mutant (and similar to the mutant obtained by the deletion of the acidic extension of APG2 (39)), raising the possibility that in addition to loss of volume, changes in the structural organization of Hsp70 after nucleotide exchange could be sensed by the SBD of Hsp110 through the extensions, and allosterically be transmitted to the NBD to stimulate ATP hydrolysis and complex dissociation. It is remarkable that, also in this case, evolution may not have invented a new mechanism but rather may have just repurposed the pre-existing SBD-NBD sensing and allosteric communication.

### Role of Hsp110 as a NEF

While the NEF activity of Hsp110s has already been documented (23, 52, 63), here we have shown that it becomes even more relevant when ATP is limiting and the ADP concentration increases, possibly being in excess of ATP. The presence of NEFs in general reduces the inhibition due to ADP and, in the case of Hsp110, can even favor the disaggregation reaction when, as in cells, hundreds of micromolar of ADP are present alongside millimolar of ATP. This NEF-driven increased resistance to the inhibition by high ADP concentrations is expected to be crucial in starving or quiescent bacteria and yeast, when ATP is not in excess. Dedicated studies are necessary in the future to elucidate how NEFs make the action of Hsp70s more resistant to increasing ADP concentrations. and NEFs may increase the affinity of Hsp70s for the increasingly rare ATP molecules.

### Molecular mechanism of action of Hsp110s

All these considerations, taken together, bring us back to the unresolved question of the composite components behind Hsp110's mechanism of action. If Hsp110 were simply nucleotide exchange factors (NEFs), as widely thought, following nucleotide exchange, they would promote Hsp70 dissociation from the substrate, halting their disaggregation/unfolding process. Thus, their ability to enhance the action of Hsp70s seems counterintuitive. In previous work, some of the authors proposed a solution to this inconsistency, suggesting that the bulkiness of Hsp110s allows them to act as selective NEFs. Specifically, Hsp110s would target isolated, unproductive Hsp70s on aggregates, accessible due to the absence of steric hindrance from other nearby Hsp70s. By contrast, Hsp110s would not act on Hsp70 clusters, which were thought to be the disaggregation foci, due to the dense packing of Hsp70s in these regions. In this model, Hsp110s (and similarly bulky NEFs, such as BAG1 conjugated with large moieties) would liberate inactive Hsp70s, making them available for further cluster formation, or clear space around clusters, promoting their growth and thus their disaggregation efficiency. However, this model has a flaw: Hsp70 clusters do actually form where steric hindrance is lesser, making these sites also more favorable to Hsp110 binding. This raises a new perspective: we propose that upon binding to substrate-bound Hsp70s, Hsp110s (or bulky BAG1) may increase entropic pulling forces, resulting in faster disaggregation. Since the increase of the disaggregation rate is exponential (in the manner of Kramers' theory, (64)) and larger than the increase of the nucleotide exchange rate and consequent substrate dissociation, this might indeed lead to the observed disaggregation enhancement, rather than inhibition, by Hsp110 (65). Thus, in addition to being a NEF, merely accelerating ADP to ATP exchange, Hsp110 would boost the basal disaggregation activity of Hsp70s by increasing their effective size and enhancing entropic pulling strokes during the short time following ADP release from Hsp70's NBDs, causing a deferred SBD opening and release of the substrate. This effect increases, with higher Hsp110 concentration, as long as they are sufficiently small, because more of them bind to the small fraction of substrate-bound Hsp70s. At higher concentrations, however, Hsp110s would begin binding to free Hsp70s in solution, resulting in larger complexes with a reduced ability to bind to the aggregates, with consequent inhibition.

Nucleotide exchange factors of the Hsp110 family represent an interesting evolutionary enigma. They belong to the Hsp70 family, but their presence in all eukaryotes with multiple cytosolic paralogs and one *endoplasmic reticulum* member suggests that their divergence and duplication preceded the appearance of the Last Eukaryotic Common Ancestor. Here we have highlighted how some of the transformations that have turned a Hsp70 disaggregating machinery into a NEF have likely resulted from the repurposing of pre-existing Hsp70 features, such as dimerization, ATPase activity, and its triggering by interaction sensing, and interdomain allosteric communication. We have, in the end, proposed a model for the molecular mechanism of action of Hsp110s as possible enhancers of entropic pulling forces. More work is needed to decide whether this model, or the others that have been proposed (44), is the correct one, and, from an evolutionary perspective, more work is also needed to fully understand the role of the Hsp70 homodimer, which we have proposed here to be an ancestral structural, but possibly also functional, template for the Hsp70-Hsp110 interaction.

## Experimental procedures

### Strains and plasmids

Wild-type *Ssa1*, *Sse1*, *Sis1*, *Ydj1, and* mutants were cloned in the pE-SUMO vector for propagation in *E. coli.* BL21-CodonPlus (DE3)-RIPL.

### Generating mutant proteins

Point mutations were introduced through site-directed mutagenesis PCR using Q5 Site-Directed Mutagenesis Kit (New England Biolabs, cat. no. E0554). Mutants were constructed by PCR by amplifying the selected regions with the primers listed in Table S1, and proteins used for this study are listed in Table S2.

### Proteins purification

For purification of the His10-SUMO tagged wild-type Sse1, Ssa1, Sis1, Ydj1, and mutants, were expressed and purified from *E. coli* BL21-CodonPlus (DE3)-RIPL cells with IPTG induction (final 0.5 mM for Ssa1 and Sse1 and 0.2 for Ydj1, Sis1, and mutants) at 18 °C, overnight. Briefly, cells were grown in LB medium + ampicillin at 37 °C to OD600 ∼0.4 to 0.5. Protein expression was induced by the addition of 0.5 mM IPTG for 3 h. Cells were harvested, washed with chilled PBS, and resuspended in buffer A (20 mM Tris-HCl pH 7.5, 150 mM KCl, 5% glycerol, 2 mM DTT, 20 mM MgCl2) containing 5 mM imidazole, 1 mg/ml Lysozyme, 1 mM PMSF for 1 h. Cells were lysed by sonication. After high-speed centrifugation (16,000 rpm, 30 min/4 °C), the supernatant was loaded onto a gravity flow-based Ni-NTA metal affinity column (2 ml beads, cOmplete His-Tag Purification Resin from Merck), equilibrated and washed with 10 column volumes of buffer A containing 5 mM imidazole. After several washes with high salt buffer A (+150 mM KCl, 20 mM Imidazole and 5 mM ATP), N-terminal His10-SUMO (small ubiquitin-related modifier) Smt3 tag was cleaved with Ulp1 protease (2 mg/ml), 300 μl, added to beads with buffer (20 mM Tris-HCl pH 7.5, 150 mM KCl, 10 mM MgCl2, 5% glycerol, 2 mM DTT). Digestion of His10 Smt3 was performed on the Ni-NTA resin by His6-Ulp1 protease. Because of dual His tags, His6- Ulp1 and His10-SUMO display a high affinity for Ni-NTA resin and remain bound to it during cleavage reaction. After overnight digestion at 4 °C, the unbound fraction is collected (which contains only the native proteins). Proteins were further purified by concentrating to ∼3 mg/ml and applying to a size exclusion column (Superdex-200 increase, 10/30 GE Healthcare) equilibrated in buffer A containing 5 mM ATP. Pure fractions were pooled, concentrated by ultrafiltration using Amicon Ultra (Millipore), aliquoted, and stored at −80 °C. All protein concentrations were determined spectrophotometrically at 562 nm using BCA Protein Assay Kit− Reducing Agent Compatible (cat no. 23250). The purified proteins were collected, concentrated, and stored at −80 °C for further use.

### Choice of substrates: luciferase and MlucV

Two well-established chaperone substrates were used: stably preformed luciferase aggregates (Shorter-like aggregate (66)) and stably urea-preformed MlucV aggregates (6). MlucV is composed of a firefly luciferase flanked by two domains of GFP-derived fluorophores, whose fluorescent resonance energy transfer (FRET) values inform on the different states of the reported protein: already stably aggregated, transiently unfolded, or native. It allows following the conversion by the Hsp70-JDP-Hsp110 chaperone system of stably pre-aggregated substrates formed before chaperone exposure, to completely or partially unfolded intermediates, and finally to the natively refolded products of the chaperone action (6). Recovered luciferase activity was used to confirm that the chaperone did mediate polypeptide disaggregation and unfolding that subsequently led to the native refolding of the core luciferase domain in the preaggregated MlucV.

### FRET measurements and proximity ratio calculation

Ensemble relative FRET ratios were determined by measuring the maximum fluorescence emission intensities of the donor (ED) and acceptor (EA) fluorophores upon excitation of the donor at 405 nm, as described previously (67, 68). The fluorescence emission spectra of the MlucV reporter were recorded using a Tecan SPARK (Männedorf) in a 96-well microtiter plate. For donor fluorophore, excitation and emission wavelengths were at 405 nm and 493 nm with an excitation bandwidth of 20 nm. For acceptor, excitation and emission wavelengths were at 405 nm and 525 nm with an excitation bandwidth of 20 nm. To minimize direct excitation of the acceptor, donor excitation was strictly maintained at 405 nm. Background subtraction was performed using spectra obtained from buffer-only samples. Where applicable, baseline-corrected spectra were further normalized to their respective areas. All measurements were carried out in LRB refolding buffer (20 mM HEPES-KOH, pH 7.5, 150 mM KCl, 10 mM MgCl_2_) supplemented with 4 mM ATP, unless stated otherwise. Each experiment was performed in triplicate or more to ensure reproducibility. Normalized FRET ratios relative to that of native MlucV were calculated as follows:

$${FRET}_{ensemble}=\frac{E_{acceptor}}{E_{donor}+E_{acceptor}}$$

Normalized FRET efficiencies relative to that of native MlucV were calculated as follows (6, 69):

$${FRET}_{norm}=\frac{{FRET}_{ensemble}-{FRET}_{separated}}{{FRET}_{native}-{FRET}_{separated}}$$

where FRET_ensemble_ is the measured ensemble FRET efficiency, FRET_separated_ is the calculated ensemble FRET measured in a solution of separated mTFP1 and Venus (∼0.335), and FRET_native_ is the measured ensemble FRET of native MlucV (∼0.425).

A FRET value for the native MlucV is set to be 100%, and a FRET value for the unconnected fluorophores is set to be 0%. FRET values for the stable, pre-aggregated MlucV (130–170% depending on aggregation conditions) and finally, a FRET value for the unfolded luciferase in MlucV in the presence of 4.8 M urea (found to be 40% of native), and for the chaperone-bound unfolded luciferase in MlucV (found to be generally less than native).

Thus, FRET measures allowed us to follow the ATP-dependent conversion of stably aggregated polypeptide substrates formed prior to chaperone exposure, to completely or partially unfolded chaperone-bound intermediates, and finally, to the natively refolded products of the chaperone reaction (6). Recovered luciferase activity was used to confirm that the chaperone did mediate polypeptide disaggregation and unfolding that subsequently led to the native refolding of the core luciferase domain in the preaggregated MlucV. For comparison with the MlucV results, urea-pre aggregated firefly luciferase was also used as a chaperone substrate (66), and only the luciferase activity, without FRET, was used to assess the chaperone disaggregation and refolding activities.

### Luciferase and MlucV refolding assay

Luciferase and MlucV activity were measured as described previously (6, 38, 70). In the presence of oxygen, luciferase catalyzes the conversion of D-luciferin and ATP into oxyluciferin, CO2, AMP, PPi, and light. Generated photons were counted with a Victor Light 1420 Luminescence Counter from PerkinElmer (Turku, Finland) in a 96-well microtiter plate format.

### ATPase assay (Malachite Green)

Colorimetric determination of Pi produced by ATP hydrolysis was performed using the Malachite Green Assay Kit (Sigma-Aldrich) and as described previously (71). Several concentrations of Hsp70 (Ssa1), JDPs (Ydj1, Sis1), or mutants were mixed with or without substrate (200 nM of pre-aggregated luciferase) and with 1 mM of ATP and incubated for 1 h at 25 °C. 8 μl of each sample was taken and put inside a 96-Well plate with 72 μl of H2O. A 20-μl volume of Malachite Green reaction buffer was added, and the samples were mixed thoroughly and incubated at 25 °C for 30 min before measuring at 620 nm on a plate reader (Tecan SPARK). The rate of intrinsic ATP hydrolysis was deduced by subtracting the signal from ATP in the absence of a chaperone.

### Phylogenetic tree

Maximum likelihood phylogenetic trees were generated by IQ-TREE2 (72), using JTT distance matrix and NJ/BioNJ initial tree. Trees were rooted at midpoint and made with iTOL (73).

## Data availability

The data underlying this article are available in the article and in its online supplementary material.

## Supporting information

This article contains supporting information (34, 51, 74, 75, 76, 77).

## Conflict of interest

The authors declare that they have no conflicts of interest with the contents of this article.
