# Supplementary material for: Hsp110 nucleotide exchange factors may amplify Hsp70-disaggregation by enhanced entropic pulling
Source: J Biol Chem. 2025 Jul 4;301(8):110450. doi: 10.1016/j.jbc.2025.110450 (PMC12305717; doi:10.1016/j.jbc.2025.110450)
Supplement: Supporting information [file mmc1.docx]

**Hsp110 Nucleotide Exchange Factors may amplify Hsp70-disaggregation by enhanced entropic pulling**

Running title: Hsp110 Enhance Hsp70 by entropic pulling strokes

Mathieu E. Rebeaud^1,2^, Bruno Fauvet^1,3^, Paolo De Los Rios*^1,3^ and Pierre Goloubinoff^2^*

^1^Institute of Physics, School of Basic Sciences, École Polytechnique Fédérale de Lausanne – EPFL, 1015 Lausanne, Switzerland

^2^Department of Plant Molecular Biology, Faculty of Biology and Medicine, University of Lausanne, CH-1015 Lausanne, Switzerland

^3^Institute of Bioengineering, School of Life Sciences, École Polytechnique Fédérale de Lausanne – EPFL, 1015 Lausanne, Switzerland

*Corresponding authors: [paolo.delosrios@epfl.ch](mailto:paolo.delosrios@epfl.ch) ; [pierre.goloubinoff@unil.ch](mailto:pierre.goloubinoff@unil.ch)

**Supporting Information**

Supplementary Figures 1-6

Tables S1-S2


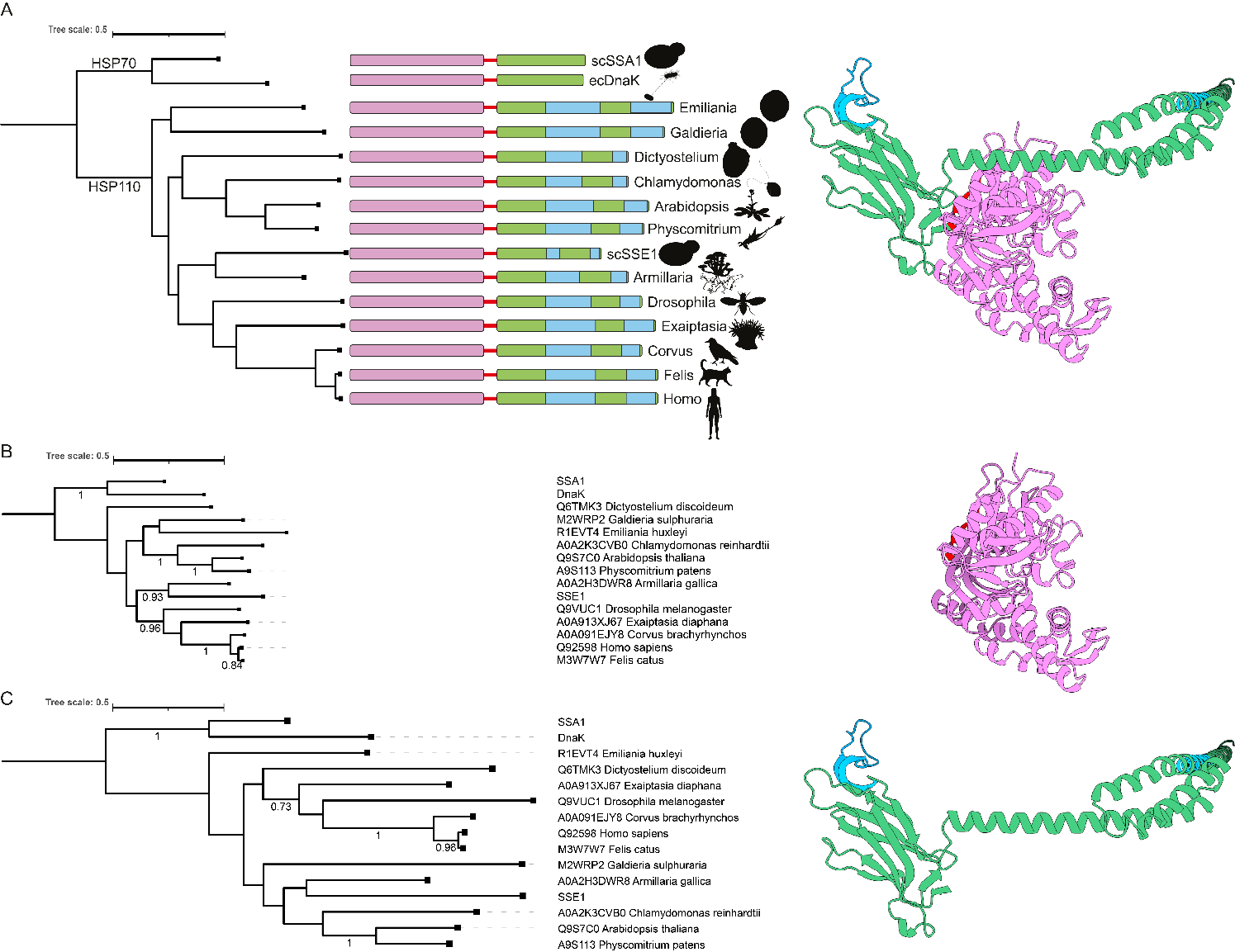


**Supplementary Figure 1: Phylogenetic trees from amino acid sequence alignments of Hsp110s from distant eukaryotes, rooted with *E. coli* DnaK and Yeast Hsp70s.** (**A**) Compared to the highly conserved Hsp70s, the SBDs of Hsp110’s contain increasingly long, variable extra loops (blue) in the SBD, which in yeast Sse1 are the shortest among all the known Hsp110s. Sequence variation shows that during the 2 billion years of eukaryote evolution, amino acid substitutions were much slower in the NBDs (**B**, Sse1 NBD in violet), than in the SBDs (**C**, Sse1 SBD in green) of both Hsp70s and Hsp110s. Bootstrap value of more than 0.7 are shown on the trees of B and C (Representative organisms pictures are from <https://www.phylopic.org>, with *Chlamydomonas* by Sergio A. Muñoz-Gómez and *Escherichia coli* by Matt Crook)


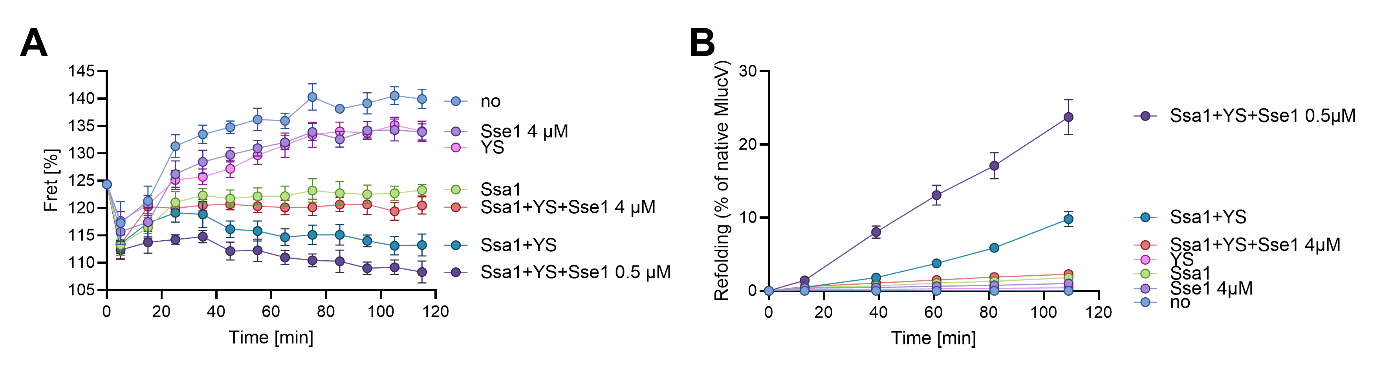


**Supplementary Figure 2: Contrary to limiting SSE1, excess Sse1 does not prevent aggregation and blocks native refolding by SSA1-SIS1.** MlucV was pre-unfolded in urea and abruptly diluted 100-fold in refolding buffer containing 4 mM ATP and after 5 min at 25°C to allow aggregation, at T=0’, 4000 nM Ssa1 or 4000 nM Sse1 was added or not (no), in the absence or presence of 1000 nM Sis1, without or with 500 nM Sse1 as indicated. FRET signals (A) and luciferase activity (B) were measured for 2 hours. In all panels, error bars represent mean ± SD (minimum of n = 3)


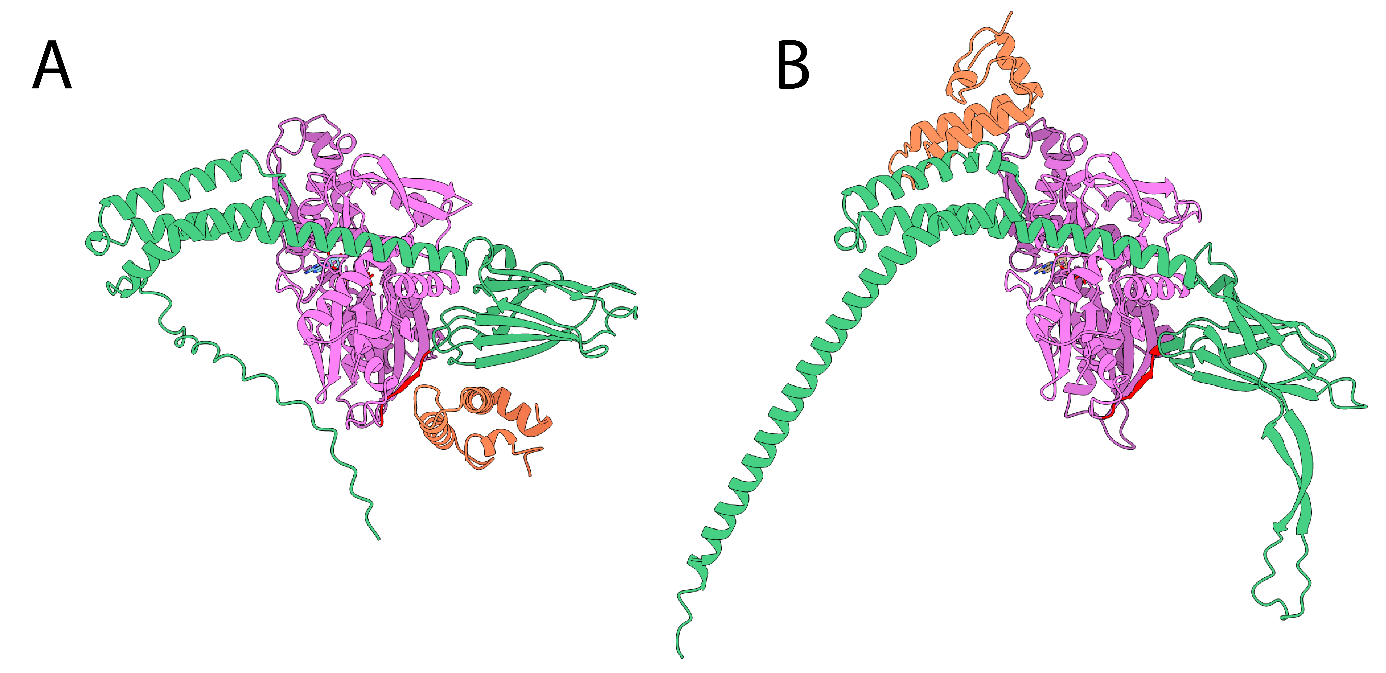


**Supplementary Figure 3:** **Sse1 has apparently lost the ability to interact with JDP.** AlphaFold 3 best prediction of the ATP-Ssa1-Sis1 (**A**) and ATP-Sse1-Sis1 (**B**) complexes. NBDs in magenta, SBDs in green, linkers in red, J-domain of Sis1 in Orange.


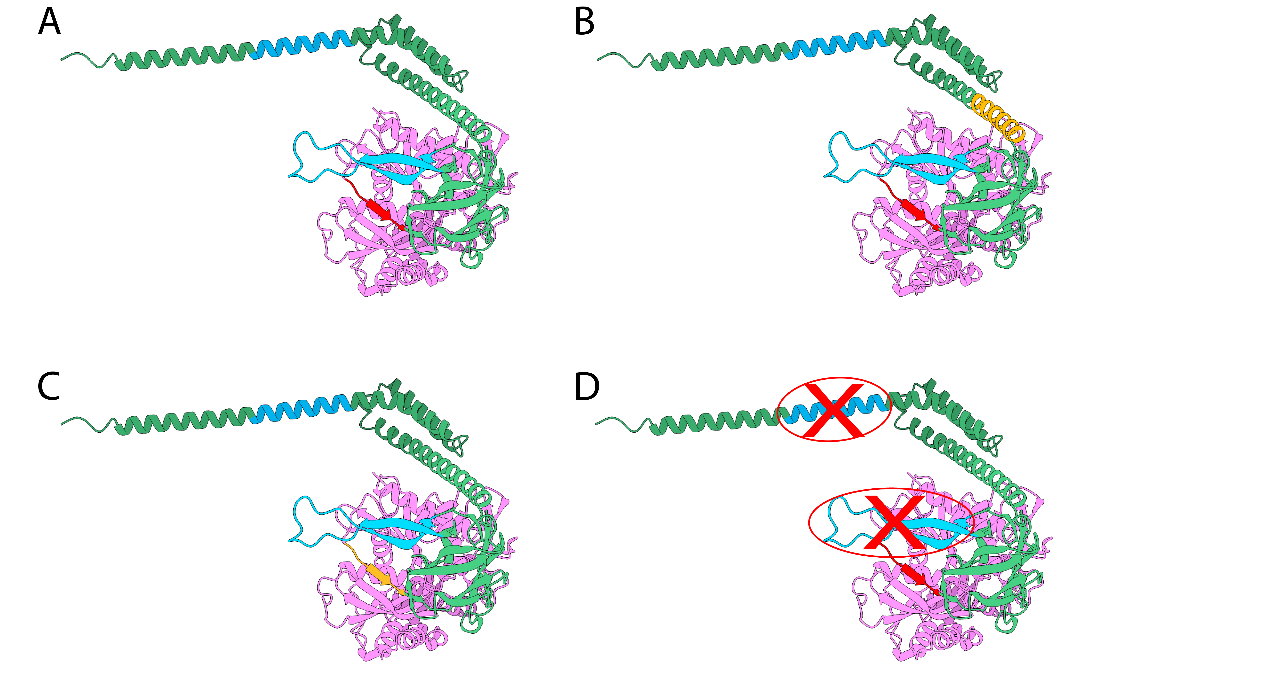


**Supplementary Figure 4. Structure of SSE1 WT with changes introduced in the mutants used in this study.** **A**: WT SSE1 AlphaFold-predicted structure (from UNIPROT: AF-P32589-F1), with NBD in purple, linker in red, SBD in green and the extensions in blue. **B**: Sse1 HM (helix mutant, orange). **C**: Sse1 LM (linker mutant, EAE, orange). **D**: Sse1 Loopout, with the extension crossed in red.


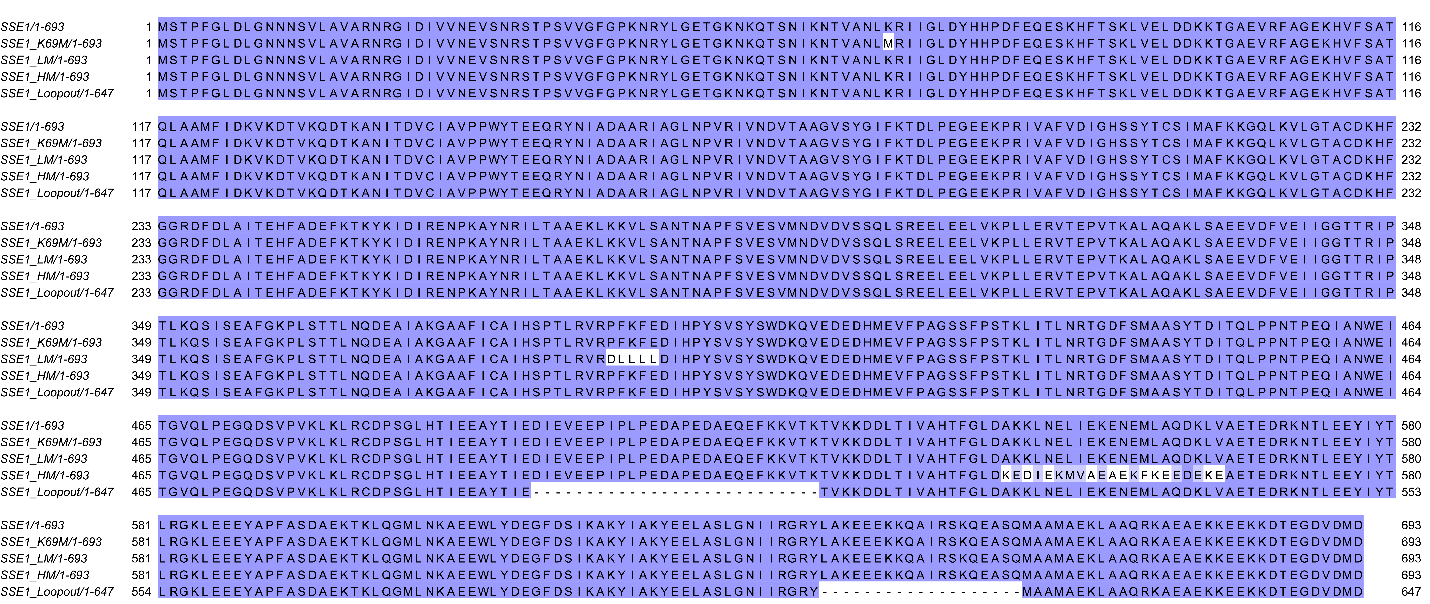


**Supplementary Figure 5. Sequence alignment of Sse1 with the mutants used in this study.** Blue, identical residues. Gaps or white, residues missing in the Loopout mutant or different in the EAE and HM mutants.


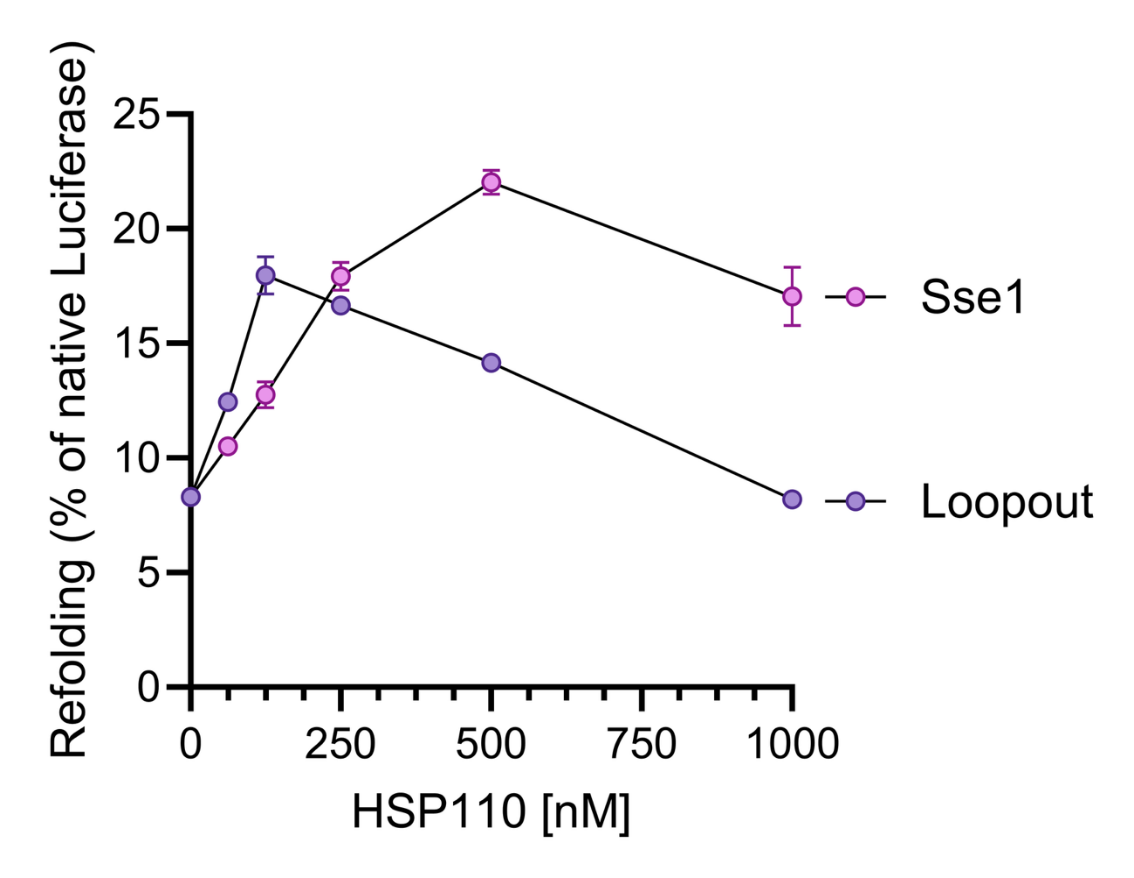


**Supplementary Figure 6. Activation profiles of Ssa1-Sis1 disaggregation and luciferase refolding by WT Sse1 and the Loopout Sse1 mutant.** Luciferase refolding yields of 200 nM preaggregated MlucV following incubation for 180 min at RT, in the presence of ATP, 6000 nM Ssa1, 1000 nM Sis1 and increasing concentrations of WT Sse1 or Loopout mutants. In all panels, error bars represent mean ± SD (n = 3)

**Supplementary Tables**

| Primer | Forward | Reverse |
| --- | --- | --- |
| Sse1HM | Insert  cacacacctttggcctagacaaagaagatatcgaaaaaatgg  Vector  gctgagacagaagaccgtaag | Insert  ttacggtcttctgtctcagcttctttttcgtcctcttc  Vector  gtctaggccaaaggtgtg |
| Sse1LM Mutagenesis | GCTGCTGGATATCCATCCTTACTCTG | AGCAGATCTCTAACTCTTAGAGTTGGAG |
| Sse1 Loopout | First loop  gtagatacATGGCTGCTATGGCTGAAAAG  Second loop  ttacactattgaaACTGTAAAGAAGGATGACTTAAC | First loop  tttacagtTTCAATAGTGTAAGCCTCTTCAATTG  Second loop  agcagccatGTATCTACCTCTAATAATGTTACC |

Table S1 Primers used in this study

| Protein | Protein family | References |
| --- | --- | --- |
| Ssa1 | Hsp70 WT | (Werner-Washburne, Stone et al. 1987) |
| Sse1 | Hsp110 WT | (Mukai, Kuno et al. 1993) |
| Sse2 | Hsp110 WT | (Mukai, Kuno et al. 1993) |
| Sis1 | JDP WT | (Luke, Sutton et al. 1991) |
| Ydj1 | JDP WT | (Caplan and Douglas 1991) |
| YS | JDP Swap | (Rebeaud, Tiwari et al. 2024) |
| Sse1HM | Hsp110 mutant | This study |
| Sse1LM (EAE) | Hsp110 mutant | This study |
| Sse1 Loopout | Hsp110 mutant | This study |
| Sse1 K69M | Hsp110 mutant | (Raviol, Sadlish et al. 2006) |

Table S2 Proteins used in this study

**References**

Caplan, A. J. and M. G. Douglas (1991). "Characterization of YDJ1: a yeast homologue of the bacterial dnaJ protein." J Cell Biol **114**(4): 609-621.

Luke, M. M., A. Sutton and K. T. Arndt (1991). "Characterization of SIS1, a Saccharomyces cerevisiae homologue of bacterial dnaJ proteins." J Cell Biol **114**(4): 623-638.

Mukai, H., T. Kuno, H. Tanaka, D. Hirata, T. Miyakawa and C. Tanaka (1993). "Isolation and characterization of SSE1 and SSE2, new members of the yeast HSP70 multigene family." Gene **132**(1): 57-66.

Raviol, H., H. Sadlish, F. Rodriguez, M. P. Mayer and B. Bukau (2006). "Chaperone network in the yeast cytosol: Hsp110 is revealed as an Hsp70 nucleotide exchange factor." EMBO J **25**(11): 2510-2518.

Rebeaud, M. E., S. Tiwari, B. Fauvet, A. Mohr, P. Goloubinoff and P. De Los Rios (2024). "Autorepression of yeast Hsp70 cochaperones by intramolecular interactions involving their J-domains." Cell Stress Chaperones **29**(2): 338-348.

Werner-Washburne, M., D. E. Stone and E. A. Craig (1987). "Complex interactions among members of an essential subfamily of hsp70 genes in Saccharomyces cerevisiae." Mol Cell Biol **7**(7): 2568-2577.
